# The thoracoacromial artery as the lifeboat in recipient artery deficiency in complex chest wall defect reconstruction

**DOI:** 10.1016/j.ijscr.2023.108057

**Published:** 2023-03-28

**Authors:** Ali Yavari, Hojjat Molaei, Arjang Ghahremani, Omid Etemad, Hesam Amini, Shahab Rafieian

**Affiliations:** a Department of Plastic and Reconstructive Surgery, Imam Khomeini Hospital Complex, Tehran University of Medical Sciences, Qarib St, Tehran, Iran; b Department of Thoracic Surgery, Imam Khomeini Hospital Complex, Tehran University of Medical Sciences, Qarib St, Tehran, Iran

**Keywords:** Latissimus dorsi, Thoracoacromial artery, Chest wall defect, Osteoradionecrosis

## Abstract

**Introduction and importance:**

Reconstruction of chest wall defects is a complex procedure requiring an accurate understanding of the complete anatomy of the chest wall to deal with challenging defects. This report investigates the use of the thoracoacromial artery and cephalic vein as recipient vessels in a musculocutaneous latissimus dorsi free flap to cover the large chest wall defect resulting from post-radiation necrosis for breast cancer.

**Case presentation:**

A 25-year-old woman with established necrotic osteochondritis of the left side ribs following radiotherapy in breast cancer management was admitted for reconstructing the violated chest wall. The contralateral latissimus dorsi muscle was selected as an alternative to the previously used ipsilateral muscle. The thoracoacromial artery was the only one available as a recipient artery with a successful outcome.

**Clinical discussion:**

Breast cancer is the most common indication for radiotherapy. Osteoradionecrosis can present months to years after radiation with deep ulcers and major bone destruction with soft tissue necrosis. Large defect reconstruction is sometimes challenging due to lack of recipient artery and vein because of previous unsuccessful interventions. Thoracoacromial artery and its branches can be recommended as a good alternative recipient artery.

**Conclusion:**

The Thoracoacromial artery may aid surgeons in achieving successful anastomoses in difficult thoracic defects.

## Highlights

• Osteoradionecrosis (ORN) of the chest wall is a rare complication that occurs after breast
• Reconstruction of chest wall defects is a complex procedure
• 25-year-old woman with established necrotic osteochondritis of left side ribs the following Radiotherapy
• The thoracoacromial artery and its branches can be recommended as a good alternative recipient artery for patients like ours who have had many unsuccessful surgeries and have not numerous options to use
• The latissimus dorsi flap is a workhorse and suitable flap for large defects of the chest

## 1 Introduction

Osteoradionecrosis (ORN) of the chest wall is a rare complication that occurs after breast radiotherapy [1]. Radiotherapy is used following surgery to eradicate the residual tumoral cells but can cause damage to adjacent bones and soft tissue [2]. Management includes radical excision and covering the defect with a workhorse flap [3], [4], [5], [6]. Similar cases treated with radical excision and coverage with an abdominal flap based on a perforator have been reported.

A case involving underlying ribs following adjuvant whole breast radiotherapy and the following unsuccessful surgery to cover the defect with a pediculated ipsilateral latissimus dorsi flap, resulting in a large chest wall defect is presented. The defect was covered by using the thoracoacromial artery and cephalic vein as recipient vessels for the musculocutaneous latissimus dorsi free flap. The thoracoacromial artery is a trunk from the second part of the axillary artery that descends between two pectoralis muscles [7]. This work has been reported in line with the SCARE criteria [8].

## 2 Presentation of case

**Fig. 1:**
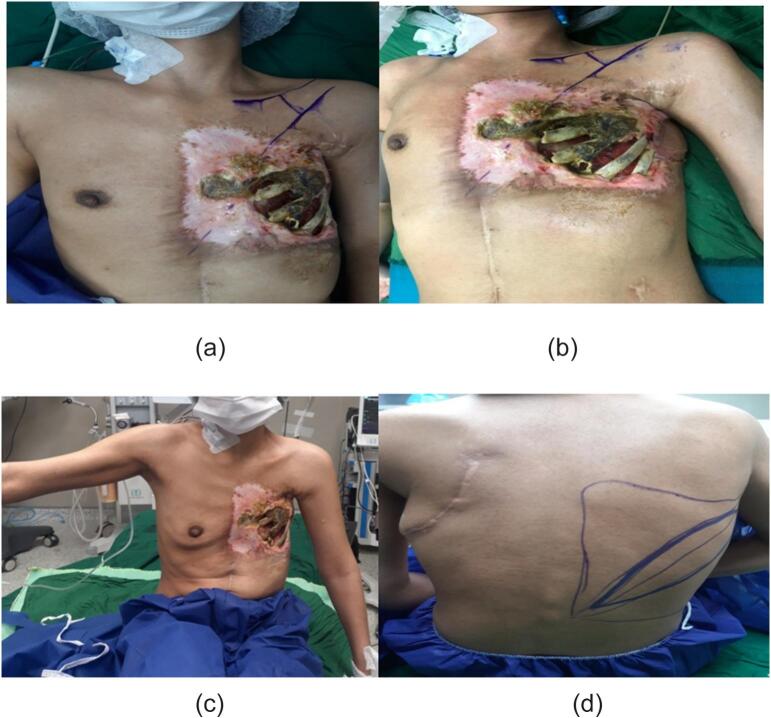
Pre-operative pictures of the patient and thoracoacromial artery direction found by doppler. In c picture restricted arm abduction is apparent.

**Fig. 2:**
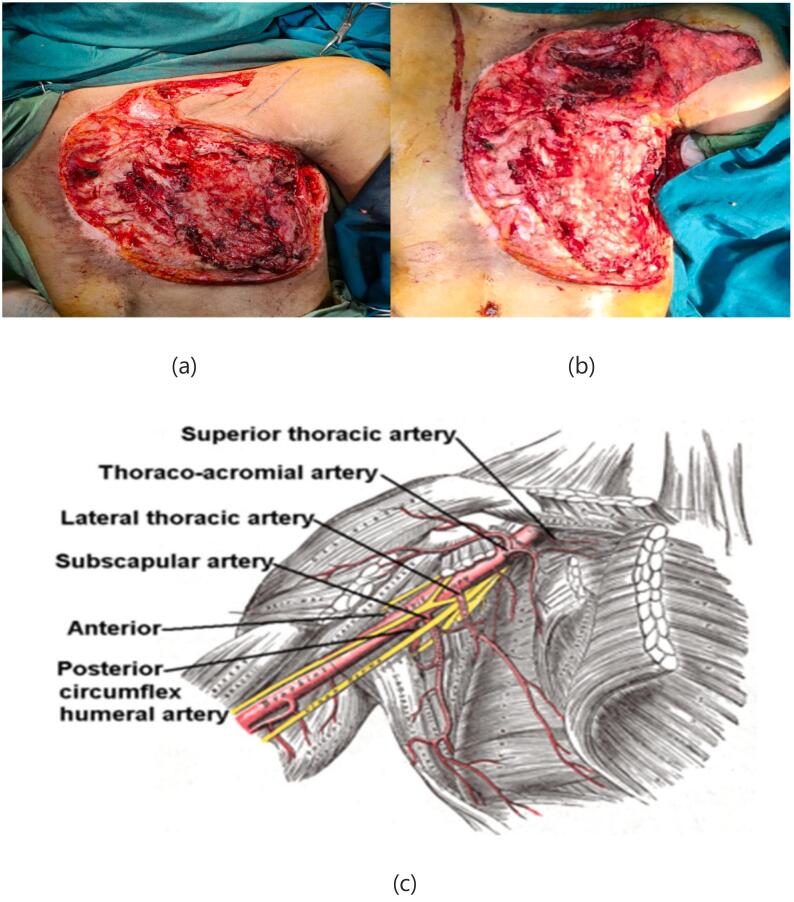
Chest defect after radical debridement, in b picture can see clavicle partial resection to access the artery, c picture shows the schematic view of thoracoacromial branch.

**Fig. 3:**
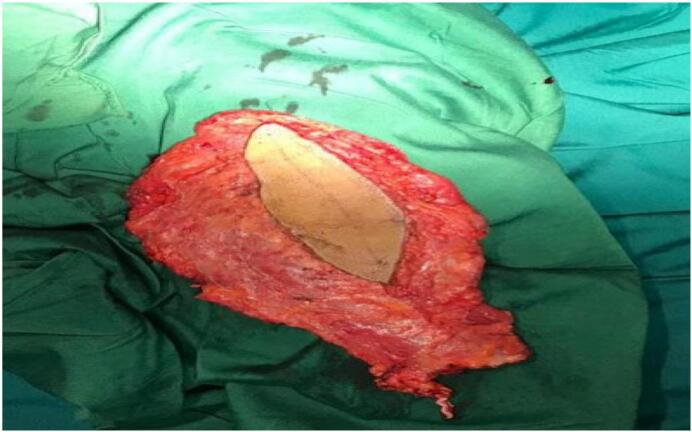
Harvested free latissimus dorsi flap.

**Fig. 4:**
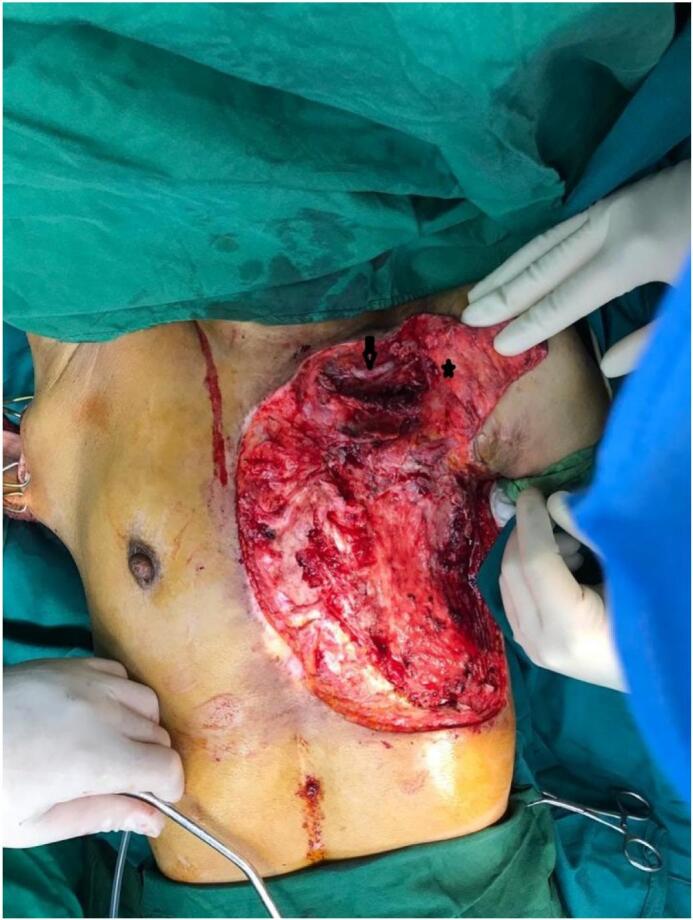
Arrow shows subclavian artery. Star shows pectoral branch of thoracoacromial artery and anastomosis.

**Fig. 5:**
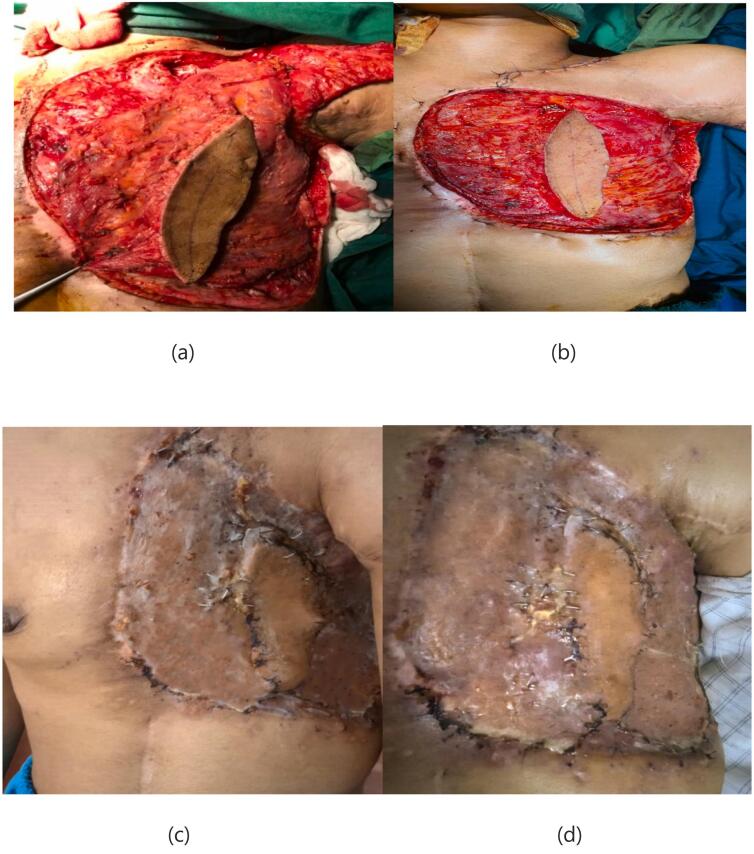
Defect during and after flap inset, can see the final result after one month in picture c.

A 25-year-old female presented with a large chest wall defect and exposed rib bones, with purulent discharge through the sinuses and peripheral pigmented skin measuring 25 cm by 30 cm on the left chest wall, resulting from ORN of the underlying second to sixth ribs and involvement up to the pleura and right atrium. The patient underwent a modified radical mastectomy two years ago, followed by whole chest wall radiotherapy for ductal carcinoma of the left breast. The patient had an unsuccessful operation in which the ipsilateral latissimus muscle (through the upper transverse incision) was used to cover the defect. Due to fibrosis at the origin of the pectoralis major muscle, arm abduction was limited. She was referred from another country and her past medical history was unclear. Her tumor stage, radiation type and dosage and tumor stage were imprecise and vague. She had no significant history of medical history other than breast cancer. The perspective of treatment was given to the patient. She accepted the risk of surgery and the final cosmetic result. Due to her unclear history and for more evaluation of the chest wall wound, a biopsy was taken from the chest wall to rule out the possible malignancy. The pathology report revealed ORN and no malignancy. Her abdominopelvic CT scan, chest CT scan brain MRI and bone scan were normal and had no evidence of metastasis. Internal mammary arteries were not suitable due to radiotherapy damage. Debridement of the infected tissues and covering the defect with a contralateral latissimus dorsi free flap was planned. The pectoral branch of the thoracoacromial artery was traced by Doppler ultrasonography (Fig. 1). After an anesthesiology consultation, she was planned for surgery. The patient's vital signs were all within the normal range (blood pressure 110/75 mm Hg, pulse rate 70 bpm, respiratory rate 18 rpm, temperature 37 °C, blood oxygen saturation 95 %). The BMI was 18.7. Lab data were within normal ranges (Hb: 14.1, Plt: 221,000, WBC: 7.23, ALT: 18, AST: 41, ALKP: 39 INR: 1). After radical debridement by the thoracic surgery team in supine position, a defect measuring 45 ∗ 35 cm (almost a left hemithorax defect) was created (Fig. 2). Partial clavicle resection for better vascular dissection and exposure was done by the thoracic surgeons, and then the recipient artery and vein were explored. In the second step of surgery after dissection through the remnant of the pectoralis major muscle by the plastic surgery team, the pectoral branch of the thoracoacromial artery was found and isolated (Fig. 2), and during the dissection to the axillary vein, the cephalic vein before connecting the axillary vein was found as the recipient's vein. In the third stage, a contralateral musculocutaneous latissimus dorsi free flap based on thoracodorsal vessels was harvested (Fig. 3). In the final stage, Prolene 9/0 was used for arterial anastomosis, and 8/0 prolene was used for venous anastomosis (Fig. 4). All anastomoses were done by loop glasses with 4× magnification. The thoracodorsal artery to the pectoral branch of thoracoacromial artery and the thoracodorsal vein to the cephalic vein were anastomosed end to end by parachute technique. A narrow strip, 6 cm wide of skin with muscle was used to make harvesting and monitoring of the free flap easier. The donor site defect was closed primarily with two suction drains in the axilla and chest wall. We used skin graft for better coverage of the remaining defects. Grafts were covered with dressing which were removed on the 6th day. The postoperative order was heparin 4000 IU subcutaneously, intravenous cephazolin 1 g QID, clindamycin 600 mg 600 MG TDS and morphine 3 mg TDS for 5 days. The flap was monitored regularly for vascularity. Drains were removed on postoperative days 7 and 14 and the patient was discharged on the 8th day. After discharge, the patient followed up with a healthy flap in the postoperative period (Fig. 5).

## 3 Surgical anatomy

The thoracoacromial artery arises from the axillary artery. It divides into four branches: pectoral, acromial, clavicular, and deltoid. The pectoral branch descends between two pectoralis muscles perpendicular to a line drawn from the acromion and the xiphoid from the medial one-third of the clavicle [7] (Fig. 1). The cephalic vein is the lateral superficial upper extremity vein that drains the radial side of the hand, forearm, and arm to the axillary vein [9].

Latissimus dorsi is a broad, workhorse type V muscle that occupies a major part of the lower and posterior part of the thorax. Its major blood supply is from the thoracodorsal pedicle superiorly and the lumbar and thoracic perforators medially [10], [11], [12], [13]. Muscles can survive based on each pedicle mentioned. To harvest free flap, the thoracodorsal pedicle is divided up into the axillary vessels. The origin of the flap is the thoracolumbar fascia inferiorly and the intertubercular groove of the humerus superiorly. In this case, because of a large defect, we needed all parts of the muscle to cover all the hemithorax (Fig. 3).

## 4 Discussion

Breast carcinoma is the most common indication for chest wall irradiation, which results in post-radiation changes in the chest wall and mediastinum [1]. Radiation-induced toxicity may cause tissue inflammation and necrosis in the chest wall through microvascular injury, inflammation, and infection [2]. Patients with ORN can present with different symptoms, ranging from occult disease to deep ulcers and major bone destruction with soft tissue necrosis, months to years after radiotherapy [14]. One of the most challenging surgeries is full-thickness resection of the chest wall with reconstruction. The loss of the chest wall can cause difficult respiration and infection. The presence of necrotic bone and soft tissue with a discharging sinus in our case required radical debridement, and to avoid injury to the right atrium of the heart during debridement of necrotic tissue, the debridement led to exposure of the pericardium and right lung. Different flaps have been used to cover similar large defects by colleagues, like the pediculated TRAM flap, the pediculated latissimus flap, the perforator base abdominal flap, etc. Our patient's defect after debridement was 35 × 45 cm and required a large tissue covering, which led us to raise a large flap. Due to previous surgery, the ipsilateral latissimus muscle could not be used, and due to a low BMI, cachexia, and previous abdominal surgery, abdominal flaps were not suitable, so we decided to use the contralateral latissimus dorsi flap. After Doppler sonography of axillary-derived vessels, a thoracoacromial artery was found and its pectoral branch was used as the recipient artery. During dissection near the axillary vein, the cephalic vein was found as the recipient before attaching the axillary vein with a good lumen and good backflow. Now the thoracoacromial artery and its branches can be recommended as a good alternative recipient artery for patients like ours who have had many unsuccessful surgeries and have not numerous options to use (Fig. 2). There are only few reports about using latissimus dorsi free flap. Most of the previous reports are reconstruction of chest wall with pediculated latissimus dorsi [15]. In one of the largest reviews, Chang et al. reviewed 113 patients who underwent 157 musculocutaneous or muscle flap reconstructions for chest wall defects. The most common diagnoses were breast cancer and sarcoma, and the majority of patients underwent only 1 operation. Only 11 % of them required free tissue transfer [16]. As studies demonstrate, there are a few experiences on using free latissimus dorsi flap.

## 5 Conclusion

ORN of the chest wall is a rare complication that occurs after breast radiotherapy. The latissimus dorsi flap is a workhorse and a suitable flap for large defects of the chest wall, and as a free flap, can be used to cover the contralateral side. Branches of an axillary artery like a thoracoacromial artery can be used as the recipient's vessel, and its lumen size and diameter are good for microscopic anastomoses. The cephalic vein is also a good candidate to be used as a recipient's vein in microscopic surgery.

## Consent

Written informed consent was obtained from the patient for publication of this case report and accompanying images. A copy of the written consent is available for review by the Editor-in-Chief of this journal on request.

## Ethical approval

This case report does not hold any personal information leading to the identification of the patient. Therefore, it is exempted from ethical approval.

## Funding

N/A.

## Guarantor

Amini Hesam.

## CRediT authorship contribution statement

Amini H: Conceptualization, Writing original draft, Investigation, Resources, Review.
Shahab Rafieian: Methodology, Validation, Supervision, Writing - Review and editing.
Ali Yavari, Hojjat Molaei, Arjang Ghahremani, Omid Etemad: Writing original draft, Resources.

## Conflict of interests

N/A.
